# Population Size and Decadal Trends of Three Penguin Species Nesting at Signy Island, South Orkney Islands

**DOI:** 10.1371/journal.pone.0164025

**Published:** 2016-10-26

**Authors:** Michael J. Dunn, Jennifer A. Jackson, Stacey Adlard, Amanda S. Lynnes, Dirk R. Briggs, Derren Fox, Claire M. Waluda

**Affiliations:** 1 British Antarctic Survey, Natural Environment Research Council, Madingley Road, Cambridge CB3 0ET, United Kingdom; 2 International Association of Antarctica Tour Operators, 320 Thames Street, Suite 264, Newport, Rhode Island, 02840, United States of America; Friedrich-Schiller-Universitat Jena, GERMANY

## Abstract

We report long-term changes in population size of three species of sympatrically breeding pygoscelid penguins: Adélie (*Pygoscelis adeliae*), chinstrap (*Pygoscelis antarctica*) and gentoo (*Pygoscelis papua ellsworthii*) over a 38 year period at Signy Island, South Orkney Islands, based on annual counts from selected colonies and decadal all-island systematic counts of occupied nests. Comparing total numbers of breeding pairs over the whole island from 1978/79 to 2015/16 revealed varying fortunes: gentoo penguin pairs increased by 255%, (3.5% per annum), chinstrap penguins declined by 68% (-3.6% per annum) and Adélie penguins declined by 42% (-1.5% per annum). The chinstrap population has declined steadily over the last four decades. In contrast, Adélie and gentoo penguins have experienced phases of population increase and decline. Annual surveys of selected chinstrap and Adélie colonies produced similar trends from those revealed by island-wide surveys, allowing total island population trends to be inferred relatively well. However, while the annual colony counts of chinstrap and Adélie penguins showed a trend consistent in direction with the results from all-island surveys, the magnitude of estimated population change was markedly different between colony wide and all island counts. Annual population patterns suggest that pair numbers in the study areas partly reflect immigration and emigration of nesting birds between different parts of the island. Breeding success for all three species remained broadly stable over time in the annually monitored colonies. Breeding success rates in gentoo and chinstrap penguins were strongly correlated, despite the differing trends in population size. This study shows the importance of effective, standardised monitoring to accurately determine long-term population trajectories. Our results indicate significant declines in the Adélie and chinstrap penguin populations at Signy Island over the last five decades, and a gradual increase in gentoo breeding pairs.

## Introduction

The three species of pygoscelid penguin, Adélie, (*Pygoscelis adéliae*), chinstrap (*Pygoscelis antarctica*) and gentoo (*Pygoscelis papua*) breed sympatrically in the West Antarctic Peninsula (WAP) and Scotia Sea, including on the South Shetland Islands, South Sandwich Islands and South Orkney Islands, [1, 2] where together they constitute more than 90% of the avian biomass, excluding South Georgia [3]. In the South Orkney Islands, including Signy Island, a previous survey estimated Adélie, chinstrap and gentoo (sub-species *ellsworthii*) breeding pairs to number 200,000–300,000, c. 600,000 and 5000–10,000 pairs respectively [1], and more recently Lynch and La Rue [4] estimated the total number of Adélie penguin pairs in the same area to be approximately 190,500.

Recent studies using data collected from a number of different sites have provided clear evidence of penguin population changes across the WAP and Scotia Sea: in particular Adélie penguin numbers are in decline at most locations [4–12]. A clear decline in chinstrap penguin populations across the same region has also been established [6, 8, 9, 11–13]. However gentoo penguins, despite declining or showing a high degree of variability in the East Antarctic Peninsula [12] have otherwise shown an opposing response to the other two species with the majority of surveyed populations either remaining constant or increasing and expanding southwards [6, 8, 11, 12, 14–16].

Changes in penguin population size have been suggested as a useful indicator of ecosystem change [4, 7, 9, 10, 13, 17–21]. The Commission for the Conservation of Antarctic Marine Living Resources (CCAMLR) monitors a number of avian and mammal krill predator species including Adélie, chinstrap and Gentoo penguins across a circum-polar area, monitoring the health of the Antarctic marine ecosystem using, amongst other parameters, penguin abundance. Such indicators are particularly valuable in the WAP and Scotia Sea as this is also a region of rapid environmental change [8, 15, 22–24]. However, if we are to understand how penguin populations can be used as ecosystem indicators, it is important to understand those mechanisms that operate at local scales and that can affect individual colonies. Whilst it is important to utilise all available information relevant to estimating the number of pairs of penguins at a particular site [25] local scale datasets do not necessarily reflect changes at other sites or indeed at a wider regional scale [12, 14]. In addition, inconsistencies and biases in long-term trends can be caused by the often opportunistic timing of censuses. Indeed, if corrections are not applied for previous breeding failure this can reduce the reliability of data from population surveys carried out late in a given season [11, 26]. Nevertheless census counts, particularly if corrected for bias created by late sampling [25], provide an important temporal and spatial dataset for use in population analyses.

Adélie, chinstrap and gentoo penguins breed sympatrically at Signy Island. Previously, Forcada et al. [8] reported abundance changes within selected Adélie and chinstrap colonies and all gentoo colonies over a 26 year period at Signy Island from 1978 to 2004. They reported breeding pair declines in Adélie and chinstrap colonies and increases in gentoo colonies, occurring in parallel with a reduction in regional sea ice extent and long-term warming. Whether this decline was reflected in numbers across the rest of the island penguin populations (in the case of Adélie and chinstrap breeding pairs), and whether the population trends reported for all three species have persisted to the present day remained unknown.

Here, we present a 38-year dataset on numbers of Adélie and chinstrap breeding pairs and breeding success in a series of colonies monitored annually at Signy Island in the austral summers from 1978/79 to 2015/16. We compare these data with comprehensive all-island pair censuses carried out at the same location between 1978/79 and 2015/16 at approximately decadal intervals (Adélie and chinstrap penguins) and annually (gentoo penguins) and also with historical records from both Signy Island and Laurie Island, also in the South Orkney archipelago. Additionally, we evaluate trends and variability in these counts for comparison with other populations across the Antarctic Peninsula and Scotia Sea regions.

## Materials and Methods

### Study site and species

**Fig 1:**
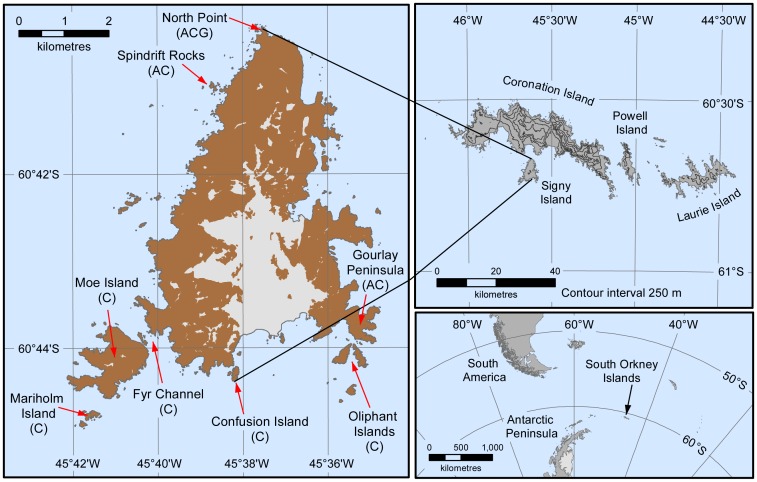
Location of South Orkney Islands, and distribution of penguin study colonies on Signy Island. Legend: A = Adélie, C = chinstrap and G = gentoo.

The study took place at Signy Island, South Orkney Islands (60°42΄S, 45°36΄W, Fig 1). At this site Adélie and chinstrap penguins breed in colonies varying in size from 15 to >2,000 pairs. Chinstrap colonies are located adjacent to those of Adélies at the south east (Gourlay Peninsula) and north of the island (North Point, Fig 1). Chinstrap penguins also nest on the south west coast (Fyr Channel) and on several offshore islands (Oliphant, Confusion, Moe and Mariholm). The entire gentoo penguin population is located at North Point (Fig 1).

### Survey methods

All surveys consisted of direct ground counts carried out by experienced observers, following the methods established by the CCAMLR Ecosystem Monitoring Programme (CEMP) [27]. Each colony was defined as a distinct assemblage of breeding pairs discrete from its neighbours and was surveyed either by marking each nest to indicate a counted pair, or observationally from the periphery of a colony using a tally counter. In the case of the latter and to ensure consistency, surveys were repeated at least three times until the count totals were within 10%, and the mean count used in further analyses. When surveying large colonies, digital photographs were taken from surrounding vantage points and breeding pairs counted from these images. Where possible, all pair counts were carried out one week after the peak of egg-laying, and fledgling counts took place once all chicks had entered crèche [27]. For Adélie and chinstrap penguins, data from designated chronological study colonies at Signy Island were used to determine the optimal count dates [27]. From 2006/07 the positions of colonies were recorded with a hand-held Global Positioning System or GPS (Garmin GPS 60), as per Waluda et al [28]. Breeding pair surveys were carried out during the incubation period for all species: Breeding birds rarely left their nests whilst being surveyed and those that did returned almost immediately.

In total, nine Adélie, eleven chinstrap and ten gentoo colonies were surveyed annually between 1978/79 and 2015/16. This included all gentoo penguin breeding pairs present on Signy Island each year. All-island breeding pair surveys of Adélie penguins were limited to approximately decadal surveys in 1978/79, 1987/88, 1994/95, 2005/06 and 2015/16, with surveys of chinstrap penguins taking place during 1978/79, 1987/88, 1994/95 and 2009/10. Chinstrap penguin breeding populations on Moe, Mariholm and Oliphant Islands, (Fig 1) were also included in all the chinstrap entire island breeding surveys except 2009/10, when inaccessibility prevented survey work.

Breeding success was calculated as annual number of chicks counted immediately prior to fledging, divided by the number of adult pairs [27]. Breeding pair counts and breeding success were plotted for all sub-colonies alongside all-island surveys. Linear regressions of pair counts over time were conducted for each species using sub-colony counts utilising the *lm* function in Program R (R Core Team 2015). Correlations between species breeding success rates were also investigated, using Pearson’s product moment correlation coefficient (*cor*.*test* in Program R).

Our research was approved by the British Antarctic Survey Animal Ethics Review Committee, and permission was granted by the British Foreign and Commonwealth Office on behalf of HM Secretary of State, under section 12 and 13 of the Antarctic Act, 1994, 2013. All relevant data are available within this paper.

## Results

### Population trends

**Fig 2:**
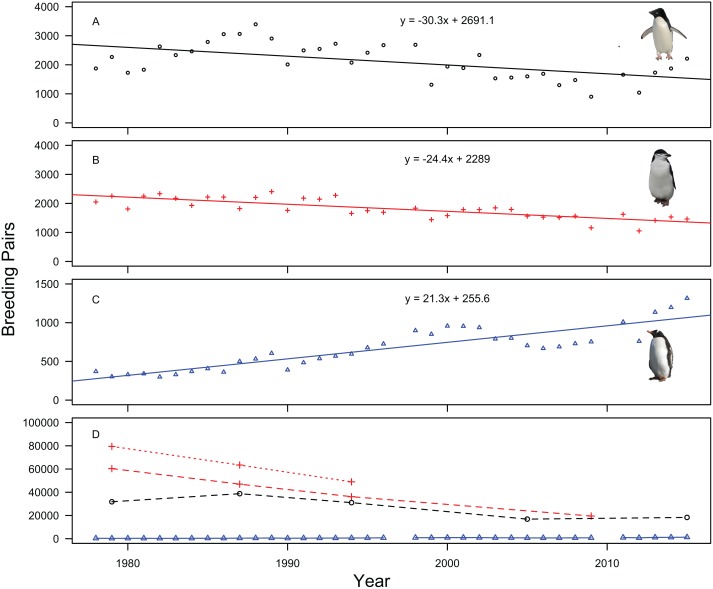
Trends in total number of penguin breeding pairs on Signy Island, 1978/79-2015/16. Legend: Panels A to C show annual counts of Adélie (9 colonies), chinstrap (11 colonies) and gentoo (all 10 Signy Island colonies) penguin breeding pairs, with accompanying linear regression values. Panel D shows all-island counts, with each species colour coded as shown in individual panels A to C. Note: chinstrap (including offshore islands) = red crosses and dashed line, chinstrap (omitting offshore islands) = red crosses and dotted line.

Between 1978/79 and 1987/88 the total number of breeding pairs of Adélie penguins present on Signy Island increased by 21.9% from 31,807 to 38,774 (+2.2% per annum), followed by a decline of 20% to 31,067 pairs (-3.1% per annum) to 1994/95. Adélie numbers declined by a further 46% to 16,872 pairs in 2005/06 (-5.4% per annum) and increased by 8.7% to 18,333 (+0.8% per annum) in 2015/16 (Fig 2, Table 1). Overall the population has experienced a 42% decline (-1.5% per annum) over the 38 year period. Annual counts from nine Adélie penguin study colonies showed a similar pattern. As with the whole island surveys, the counts increased, in this case by 81.3% from 1,873 pairs in 1978/79 to a peak of 3,395 pairs in 1988/89 (+6.1% per annum), then decreased by 73% to 901 pairs in 2009/10 (-6.1% per annum) followed by an increase of 146% (+13.7% per annum) to 2,212 pairs in 2015/16 (Fig 2, Table 2). Over the study period, three Adélie penguin colonies disappeared completely. A linear regression of pair counts through time showed a strong fit to these data, with adjusted R^2^ indicating that 30% of the variance in the data can be explained by a steady population decline of ~30 breeding pairs per year (Fig 2).

**Table 1:** Pairs of Adélie, chinstrap and gentoo penguins counted at Signy Island and offshore islands, 1947/48–2015/16.

Season	Total breeding pairs								
	Adélie Counted nests	Adelie Estimated breeding pairs	Date	Chinstrap counted nests	Chinstrap estimated pairs	Date	Gentoo counted nests	Date	Source
1947/48		10500	21 Nov. 1947	n/a	9000		300	20 Nov. 1948	Croxall & Kirkwood [29]
1957/58	n/a	Partial survey		n/a			314	06 Jan. 1958	Croxall & Kirkwood [29]
1963/64	n/a	n/a		n/a			200	20 Jan. 1964	Croxall & Kirkwood [29]
1976/77	n/a	n/a		n/a			255	12 Dec. 1976	Croxall & Kirkwood [29]
1978/79	31,807	37200	13 Nov-6 Dec 1978	79,504		06–15 Dec. 1978	370	26 Nov. 1978	Croxall et al [30]
1987/88	38,774		08–10 Nov 1987	63,440		03–20 Dec. 1987	500	18 Nov. 1987	This study
1994/95	31,067		01–12 Nov 1994	48,980		06–21 Dec. 1994	595	18 Nov. 1994	This study
2005/06	16,872		15–18 Nov 2005				704	30 Nov. 2005	This study
2009/10				19,530		11–24 Dec. 2009	753	18 Nov. 2009	This study
2015/16	18333		18–22 Nov. 2015				1315	23 Nov. 2015	This study

Note: 2009/10 chinstrap penguin survey does not include all offshore islands previously surveyed.

**Table 2:** Pairs of Adélie, chinstrap and gentoo penguins recorded in study areas at Signy Island 1978/79–2015/16.

Season	Adélie breeding pairs	Date of pair count	Chinstrap breeding pairs	Date of pair count	Gentoo breeding pairs	Date of pair count
1978/79	1873	5 Dec.1978	2050	14 Dec. 1978	370	26 Nov. 1978
1979/80	2269	16 Nov. 1979	2253	08 Dec. 1979	303	19 Nov. 1979
1980/81	1726	11 Nov. 1980	1809	29 Dec. 1980	330	11 Nov. 1980
1981/82	1831	22 Nov. 1981	2250	25 Dec. 1981	341	22 Nov. 1981
1982/83	2631	19 Nov. 1982	2334	04 Dec. 1982	299	21 Nov. 1982
1983/84	2334	23 Nov. 1983	2176	08 Dec. 1983	330	23 Nov. 1983
1984/85	2464	11 Nov. 1984	1929	12 Dec. 1984	370	11 Nov. 1984
1985/86	2787	09 Nov. 1985	2219	19 Dec. 1985	407	09 Nov. 1985
1986/87	3055	07 Nov. 1986	2218	16 Dec. 1986	362	07 Nov. 1986
1987/88	3063	08 Nov. 1987	1820	07 Dec. 1987	500	18 Nov. 1987
1988/89	3395	06 Nov. 1988	2206	09 Dec. 1988	532	06 Nov. 1988
1989/90	2904	06 Nov. 1989	2405	07 Dec. 1989	605	18 Nov. 1989
1990/91	2012	04 Nov. 1990	1761	06 Dec. 1990	390	18 Nov. 1990
1991/92	2496	05 Nov. 1991	2181	06 Dec. 1991	484	04 Dec. 1991
1992/93	2546	05 Nov. 1992	2145	07 Dec. 1992	536	26 Nov. 1992
1993/94	2725	04 Nov. 1993	2277	05 Dec. 1993	568	21 Nov. 1993
1994/95	2074	08 Nov. 1994	1655	06 Dec. 1994	595	18 Nov. 1994
1995/96	2417	07 Nov. 1995	1748	07 Dec. 1995	677	18 Nov. 1995
1996/97	2676	09 Nov. 1996	1694	05 Dec. 1996	726	19 Nov. 1996
1998/99	2688	13 Nov. 1998	1836	07 Dec. 1998	898	27 Nov. 1998
1999/00	1313	11 Nov. 1999	1440	06 Dec. 1999	851	20 Nov. 1999
2000/01	1939	13 Nov. 2000	1579	11 Dec. 2000	957	25 Nov. 2000
2001/02	1888	15 Nov. 2001	1785	09 Dec. 2001	954	25 Nov. 2001
2002/03	2337	14 Nov. 2002	1786	11 Dec. 2002	937	25 Nov. 2002
2003/04	1533	15 Nov. 2003	1847	14 Dec. 2003	790	27 Nov. 2003
2004/05	1560	12 Nov. 2004	1791	11 Dec. 2004	800	25 Nov. 2004
2005/06	1601	15 Nov. 2005	1562	19 Dec. 2005	704	30 Nov. 2005
2006/07	1690	20 Nov. 2006	1527	17 Dec. 2006	668	30 Nov. 2006
2007/08	1299	25 Nov. 2007	1513	17 Dec. 2007	689	05 Dec. 2007
2008/09	1474	22 Nov 2008	1564	07 Dec. 2008	730	06 Dec. 2008
2009/10	901	17 Nov. 2009	1159	11 Dec. 2009	753	18 Nov. 2009
2011/12	1659	24 Nov. 2011.	1625	09 Dec. 2011	1009	24 Nov. 2011
2012/13	1040	3 Dec. 2012	1053	17 Dec. 2012	760	03 Dec. 2012
2013/14	1731	3 Dec. 2013	1417	19 Dec. 2013	1136	09 Dec. 2013
2014/15	1873	1 Dec. 2014	1533	19 Dec.2014	1197	01 Dec. 2014
2015/16	2212	23 Nov. 2015	1464	16 Dec. 2015	1315	23 Nov. 2015

Note: All surveys were conducted during incubation period of each species. The 1997/98 and 2010/11 seasons are missing as personnel were not present.

Chinstrap penguins have undergone a continuous decline between 1978/79 and 2009/10; not including all offshore islands they decreased by 22% to 1987/88 from 60,379 pairs to 46,982 (-2.7% per annum), further decreasing by 23% to 36,188 pairs in 1994/95 (-3.7% per annum) and decreasing by 54% to 19,530 pairs in 2009/10 (-4% per annum, Fig 2, Table 1). Overall the all-island population has experienced a 67.7% decline (-3.6% per annum) in pair numbers over the 32 year period between 1978/79 and 2009/10.

Across the 11 annually monitored chinstrap colonies numbers of breeding pairs fell by 28.6% (-0.9% per annum) between 1978/79 and 2015/16 from 2,050 to 1,464 pairs respectively (Fig 2, Table 2). This represents a decline in abundance but is much less marked than the decline recorded in the all-island count. Linear regression of pair counts through time showed a strongly significant fit, with adjusted R^2^ indicating that 63% of the variance in annual estimates is explained by a linear decline of ~24 breeding pairs per year (Fig 2). The annually monitored chinstrap penguin colonies moved through three periods of increase, each followed by decrease: pair numbers, despite fluctuations, increased by 11% between 1978/79 and 1993/94 from 2050 pairs to 2,277 pairs, (+0.7% per annum), then decreased by 37% (-7.4% per annum) to 1999/00 (1,440 pairs). There was a subsequent population increase of 28% (1,847 pairs, +6.4% per annum) until 2003/04, and a second period of decline until 2009/10 of 37% (1,159 pairs, -7.5% per annum). Finally there was a third increase of 40% in pair numbers (1,625 pairs) to 2011/12 (+18.4% per annum) before decreasing by 10% to 1,464 pairs (-2.6% per annum), in 2015/16 (Fig 2, Table 2). Over the study period, three chinstrap penguin colonies disappeared completely.

In contrast, the gentoo penguin population went through several periods of change: increasing between 1978/79 and 2000/01 by 159% from 370 to 957 pairs (+4.4% per annum), followed by a 30% decline to 668 pairs in 2006/07 (-5.8% per annum) and most recently increasing by 97% to 1,315 pairs in 2015/16, (+7.8% per annum, Tables 1 and 2). This represents a total population increase of 255% (3.5% per annum) between 1978/79 and 2015/16 (Fig 2). Linear regression strongly supports a regular increase in pair numbers through time (adjusted R^2^ = 0.77), with an island-wide increase of ~21 breeding pairs per annum (Fig 2).

### Breeding success

**Fig 3:**
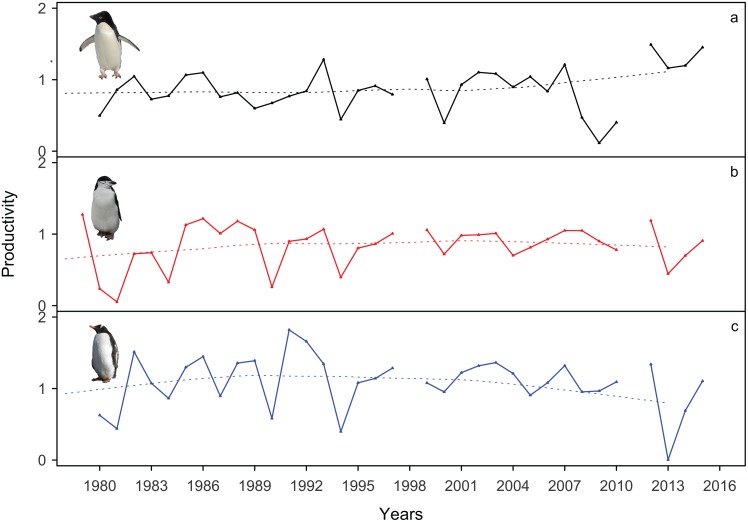
Annual penguin breeding success from 1978/79-2015/16 on Signy Island. Legend: the proportion of chicks fledged by breeding pairs (breeding productivity) from (a) Adélie, (b) chinstrap and (c) gentoo colonies. Note this data includes the entire island gentoo penguin population. Smooth trends (dashed lines) are plotted using least squares fitting of a first order polynomial for each species.

Substantial variation in breeding success was observed in all three species over the period of the study. However, in all three species the overall trend remained broadly constant: Adélie breeding success in the nine study areas varied between 5% and 74.5% (0.1–1.5 productivity, calculated as number of chicks fledged per breeding pair) over the period from 1979/80 to 2015/16, (Table 3, Fig 3). Gentoo breeding success varied between 0 and 63.5% (0.05–1.27 productivity) over the same period and chinstrap breeding success ranged from 2.5% to 63.5% (0.63–1.06 productivity) between 1978/79 and 2015/16 (Table 3, Fig 3). We found a strongly significant positive correlation between gentoo and chinstrap breeding success (correlation coefficient = 0.73). No other inter-species comparisons showed significant correlations (Fig 3).

**Table 3:** Adélie, chinstrap and gentoo penguin breeding success recorded in study areas at Signy Island 1978/79–2015/16.

Season	Adélie chicks fledged per pair	Date of chick count	Chinstrap chicks fledged per pair	Date of chick count	Gentoo chicks fledged per pair	Date of chick count
1978/79	No data	No data	1.27	16 Feb. 1979	No data	No data
1979/80	0.5	02 Feb. 1980	0.24	05 Mar. 1980	0.63	02 Feb. 1980
1980/81	0.86	28 Jan. 1981	0.05	11 Mar. 1981	0.44	28 Jan. 1981
1981/82	1.05	28 Jan. 1982	0.72	06 Mar. 1982	1.51	26 Jan. 1982
1982/83	0.73	28 Jan. 1983	0.74	No data	1.07	13 Jan. 1983
1983/84	0.77	31 Jan. 1984	0.33	06 Mar. 1984	0.86	31 Jan. 1984
1984/85	1.07	20 Jan. 1985	1.13	25 Feb. 1985	1.30	25 Jan. 1985
1985/86	1.10	23 Jan. 1986	1.22	17 Feb. 1986	1.45	23 Jan. 1986
1986/87	0.76	26 Jan. 1987	1.01	25 Feb. 1987	0.90	25 Jan. 1987
1987/88	0.82	26 Jan. 1988	1.18	21 Feb. 1988	1.36	13 Feb. 1988
1988/89	0.60	28 Jan. 1989	1.06	13 Feb. 1989	1.39	28 Jan. 1989
1989/90	0.67	26 Jan. 1990	0.26	14 Feb. 1990	0.58	26 Jan. 1990
1990/91	0.77	27 Jan. 1991	0.90	18 Feb. 1991	1.82	27 Jan. 1991
1991/92	0.84	31 Jan. 1992	0.93	17 Feb. 1992	1.66	30 Jan. 1992
1992/93	1.28	24 Jan. 1993	1.07	14 Feb. 1993	1.35	24 Jan. 1993
1993/94	0.45	30 Jan. 1994	0.40	03 Mar. 1994	0.40	23 Feb. 1994
1994/95	0.85	21 Jan. 1995	0.80	22 Feb. 1995	1.08	08 Feb. 1995
1995/96	0.91	23 Jan. 1996	0.86	14 Feb. 1996	1.14	04 Feb. 1996
1996/97	0.79	21 Jan. 1997	1.01	11 Feb. 1997	1.29	21 Jan. 1997
1998/99	1.01	15 Jan. 1999	1.06	20 Feb. 1999	1.08	21 Jan. 1999
1999/00	0.40	11 Jan. 2000	0.72	15 Feb. 2000	0.95	21 Jan. 2000
2000/01	0.93	15 Jan. 2001	0.98	16 Feb. 2001	1.22	25 Jan. 2001
2001/02	1.29	15 Jan. 2002	0.99	16 Feb. 2002	1.32	24 Jan. 2002
2002/03	1.08	21 Jan. 2003	1.01	22 Feb. 2003	1.36	02 Feb. 2003
2003/04	0.90	14 Jan. 2004	0.70	21 Feb. 2004	1.21	14 Jan. 2004
2004/05	1.04	14 Jan. 2005	0.80	19 Feb. 2005	0.91	14 Jan. 2005
2005/06	0.84	25 Jan. 2006	0.93	19 Feb. 2006	1.08	25 Jan. 2006
2006/07	1.21	18 Jan. 2007	1.05	20 Feb. 2007	1.32	18 Jan. 2007
2007/08	0.47	20 Jan. 2008	1.05	16 Feb. 2008	0.95	30 Jan. 2008
2008/09	0.11	26 Jan 2009	0.90	21 Feb. 2009	0.97	13 Jan. 2009
2009/10	0.40	15 Jan. 2010	0.78	13 Feb. 2010	1.09	15 Jan. 2010
2011/12	1.49	16 Jan. 2012.	1.19	17 Feb. 2012	1.34	16 Jan. 2012
2012/13	1.16	17 Jan. 2013	0.45	22 Feb. 2013	0.00	07 Feb. 2013
2013/14	1.20	16 Jan. 2014	0.70	13 Feb. 2014	0.69	01 Feb. 2014
2014/15	1.45	12 Jan. 2015	0.91	14 Fab. 2015	1.11	04 Feb. 2015
2015/16	1.08	15 Jan. 2016	0.77	18 Feb. 2016	1.06	26 Jan. 2016

Note: All surveys were conducted during crèche period of each species, immediately prior to fledging. The 1997/98 and 2010/11 seasons are missing as personnel were not present.

## Discussion

### Penguin population trends

The total island census data shows that the number of both Adélie and chinstrap penguin breeding pairs present on Signy Island has fallen significantly since 1978/79. Although Adélie pair numbers did initially increase during the 1980s, subsequently they have declined and despite a recent small increase between 2005/06 and 2015/16, the population has reduced by 42%. Chinstrap pair numbers have declined continuously since 1978/79, falling by 68% up to the last census in 2009/10. Interestingly, the much smaller gentoo penguin breeding population has, whilst experiencing several periods of fluctuating fortunes, nevertheless increased by 255% over the past 38 years.

Historical data from Signy Island [31, 32] suggests population increases took place in all three species up to 1978/79 from a 1947/48 estimate of 10,500 Adélie pairs, 9,000 chinstrap pairs and an actual count of 300 gentoo pairs (Table 1). This equates to increases of 203%, 783% and 23% in Adélie, chinstrap and gentoo penguins respectively. Unfortunately, a lack of information on survey effort or rigour means that these early data should be treated with caution. Nevertheless, it appears that Adélie and chinstrap penguin populations on Signy Island underwent a very large increase during a 30 year period from the mid-1940s, in contrast to the pattern of significant decline revealed in our studies from the late 1970s (chinstraps) and 1980s (Adélies) onwards. Interestingly, in contrast the gentoo population appears to have undergone a similar pattern of slow fluctuating increase during this pre-1978 period, as during the subsequent 38 years to the present. The population trajectories of these three species at Signy Island are similar to findings from other studies carried out across the Scotia Arc/WAP, indicating a pattern of decline across this region in the case of Adélie and chinstrap penguins, and an increase in gentoo penguins [9, 11–13, 15].

Elsewhere in the South Orkney Archipelago, at Laurie Island, a similar population trend has been shown for Adélie penguins, with numbers of pairs decreasing by 32% (-1.8% per annum) between 1983/84 and 2004/05 [33]. The chinstrap population trajectory on Laurie Island is less clear: as in the case of Signy Island, Coria et al. [33] suggest a large scale increase in the breeding population (384%) between 1947/48 and 1983/84, based on historical data. However, pair counts between 1983/84 and 1994/95 indicate a largely stable population increasing slightly by 1.3% (0.11% per annum [33]). The same study reported a subsequent partial survey in 2004/05 indicating a mixed pattern with some colonies increasing in pair numbers and others decreasing, although without a complete survey no clear trends could be discerned. Since the chinstrap penguin data from Signy Island bear similarities to the Laurie Island trends, albeit up to the early 1980s, future surveys at Laurie Island would be useful in establishing whether the very significant decline in chinstrap penguin pairs at Signy Island is repeated there. Ideally, additional surveys of pygoscelid populations at other breeding sites within the South Orkney Islands, using standardised methodologies, would be desirable in determining trends in population size and breeding success on a larger scale, and to compare with ongoing monitoring at Signy Island. A large-scale population survey across the whole of the South Orkney archipelago for all three pygoscelid species was carried out in 1983/84 [1]. This study estimated there to be circa 200,000–300,000 pairs of Adélie, a minimum of 600,000 pairs of chinstrap and 5,000–10,000 pairs of gentoo penguins on all islands combined. Although Lynch and LaRue [4], using ground counts and satellite imagery for the whole Antarctic region, estimated the total South Orkney Adélie penguin population to be approximately 190,500 pairs (95th percentile), no subsequent combined species survey on this scale or similar has been carried out to date.

### Population dynamics and environmental drivers

Breeding success has remained broadly similar for all three penguin species at Signy Island, as found in other populations in the Scotia Arc/WAP region [9, 10, 34]. All have experienced a series of fluctuations over time, suggesting similar annual effects on fledging success for each species, particularly given the positive correlation between gentoo and chinstrap penguin breeding success. Interestingly, Adélie penguin productivity at Signy Island has actually been highest in recent years: 75% and 73% breeding success in 2011/12 and 2014/15 or 1.49 and 1.45 créched chicks per nest, (Fig 3). In the case of Adélie penguins, Signy Island breeding success rates are comparable with and, in many cases, have a higher maximum success level than recorded elsewhere in the Antarctic ([10] and references within). Gentoo breeding success at Signy Island also compares closely with values reported elsewhere [35–37], as is the case for chinstrap breeding success [37, 38].

At a regional scale, a number of studies have revealed that in some extreme years, sea ice conditions in the South Orkney Islands has had a major influence on the numbers of penguins (particularly chinstraps) arriving to breed [30, 39, 40], and on reproductive success [41]. Forcada et al. [8] using time series data between 1978/79 and 2004/05 from the annually monitored Signy Island study colonies, found differences in responses to regional winter sea ice conditions between the pagophilic (ice-loving) Adélie and pagophobic (ice-avoiding) chinstrap penguins, concluding that variation in penguin populations reflected the balance between penguin adaptation to sea ice conditions and changes cascading from global climate forcing influencing prey availability in the foraging area of the penguins. Trathan et al. [21] noted a nonlinear response amongst chinstrap penguin pair numbers to sea-ice loss at Signy and Lynch et al. [12] found no correlation between Adélie and chinstrap population trends and changing sea-ice conditions (in November) at multiple breeding sites across the Scotia Arc and Antarctic Peninsula, suggesting additional influencing factors including over-winter juvenile survival [9, 13], and varying krill recruitment [42]. The recovery of whale species and Antarctic fur seal (*Arctocephalus gazella*) populations in the Scotia Arc/WAP region in recent decades has also been suggested as a potential source of exploitative or interference competition with foraging penguins [12, 13, 43]. Between the late 1970s and 2008 the number of Antarctic fur seals counted annually at Signy Island increased tenfold from 1,643 in 1977 to a maximum of 21,303 in 1994, with almost all animals being identified as juvenile males most likely from South Georgia [44]. The decreasing Macaroni penguin *(Eudyptes chrysolophus)* population at South Georgia has been linked to competition from increasing fur seal numbers [43] and similar competition between fur seals and penguins in the South Orkneys may be taking place.

This study finds that despite differing trends in abundance, there is in fact a very strong correlation between chinstrap (declining population) and Gentoo (increasing population) annual breeding success. This disparity between annual breeding success and actual pairs present suggests the decline in numbers of chinstrap penguins at Signy Island is unlikely to be driven by low breeding productivity or by sub-optimal breeding sites. We believe our results are consistent with increased over-winter mortality, particularly in juvenile birds as discussed in other pygoscelid population studies in this region [7, 9, 10, 12, 13]. Indeed, the similarity of our data to findings in these studies would appear to add further weight to this conclusion.

### Scale of population monitoring

Data from the annually monitored colonies on Signy Island indicated broadly similar trends in both Adélie and chinstrap penguin breeding pairs through time at colony level compared with the all-island census data between 1978/79 and 2015/16. This shows that the annual colony surveys are useful in inferring overall population trends. However, the differences in magnitude of pair trends between the annual colony counts and decadal all-island censuses indicate the limitations of small-scale counts when attempting to infer absolute abundance on a larger scale [43]. For example, the chinstrap study colonies declined across the 38 year period at a slower rate per annum and to a lesser extent than revealed by the all-island census (28.6% decline in the chinstrap study colonies as opposed to 68% island-wide). The Adélie study colonies did not show an overall decline in breeding pair numbers over the survey period (1978/79 to 2015/16), although regression of the annual pair counts supported a declining population (Fig 2). All-island censuses revealed that the population had declined by 42% during the same period. This has important implications for monitoring methodologies in general, specifically the need for supplementing frequent monitoring of small areas that can be achieved without extensive effort, with more comprehensive large-scale surveys of the same populations [45]. Although traditional direct ground count surveys, (as per CEMP standard methods [27]), on large scales require considerably more effort, they are essential if we are to avoid the risk of inaccurately inferring larger scale absolute abundance from detailed long-term studies of individual breeding sites, (see also [12, 14, 34]).

The annually monitored Adélie and chinstrap penguin colonies also revealed a series of inter-annual population fluctuations, suggesting that both species, at least on the local scale, have moved through several phases of population increase and decrease. Several Adélie and gentoo colonies entirely disappeared over the time period studied, and no new colonies were formed by any of the three species. At present it is unclear why certain study colonies have declined more rapidly than others. The substantial seasonal fluctuations in pair numbers in the Adélie and chinstrap annually monitored study colonies may reflect immigration/emigration of individual pairs at a colony-scale level to and from other breeding sites on Signy Island, as opposed to an island-wide population change, particularly as these trajectories were not reflected in the all-island surveys. However, the limited frequency of the all-island surveys prevents detection of similar trends on a larger scale.

New technology in the form of remotely-sensed satellite imagery [12, 46], digital mapping [28], unmanned aerial systems or UASs [47], and remote camera technology [48–50] all represent emerging opportunities for enabling the regular collection of large-scale population census data. However, since all of these methods have their own associated difficulties, such as obtaining cloud-free imagery from satellites [12, 46, 50] or suitable flying conditions for UASs, [47], deployment at Signy Island would require careful consideration, particularly as consistency with the long-established CEMP standard monitoring protocol [27] so far used at this site would be highly desirable.

In this study we were unable to investigate the potential influence of adverse weather conditions on population size; future use of automatic weather stations to collect meteorological data would be beneficial, particularly as previous studies at other locations have shown that stochastic environmental factors such as snow cover and air temperature can exert a spatial and temporal influence on seabird breeding success [50, 51].

## Conclusion

The populations of Adélie, chinstrap and gentoo penguins at Signy Island have changed significantly over the past five decades. Understanding the details of how such changes might be manifest in different colonies within a population, particularly with respect to individual, small-scale colonies will be essential in providing accurate data with which to test model predictions and monitor future ecosystem change. Continued, comprehensive long-term monitoring of these populations, together with future surveys of other breeding localities within the South Orkney Islands using standard methodologies will greatly assist our understanding of the large-scale processes that produce these changes, itself an important issue in the development of predictive models of penguin population status.
